# Phlebosclerotic colitis: A case report and review of the literature

**DOI:** 10.3892/etm.2014.1492

**Published:** 2014-01-20

**Authors:** YUE-LIN FANG, HO-CHI HSU, YENN-HWEI CHOU, CHIN-CHU WU, YUH-YU CHOU

**Affiliations:** 1 Division of General Surgery, Department of Surgery, Shin Kong Wu Ho-Su Memorial Hospital, Taipei 111, Taiwan, R.O.C.; 2 Department of Radiology, Shin Kong Wu Ho-Su Memorial Hospital, Taipei 111, Taiwan, R.O.C.; 3 Department of Pathology, Shin Kong Wu Ho-Su Memorial Hospital, Taipei 111, Taiwan, R.O.C.

**Keywords:** phlebosclerotic colitis, mesenteric phlebosclerosis, ischemic colitis, calcifications

## Abstract

Phlebosclerotic colitis (PC) is a rare but potentially life-threatening disease. The initial presentation may include non-specific symptoms, such as vomiting, constipation and abdominal pain; however, intestinal stenosis, gangrene and perforation may occur without appropriate management. The present report describes the case of a 56-year-old male with abdominal pain and constipation. Imaging studies revealed thread-like calcifications involving almost the entire colon, which had markedly progressed over a three-year period, and changes consistent with colonic ischemia. Angiography revealed decreased blood flow in the mesenteric veins. The patient underwent emergent subtotal colectomy, and pathological examination revealed gangrene of the colon and calcifications of the mesenteric veins. The patient’s postoperative course was uneventful. In conclusion, PC is a potentially life-threatening condition that may be diagnosed by the presence of serpentine calcifications on imaging studies. Management depends on the severity of the disease, ranging from close follow-up to prompt surgical intervention.

## Introduction

Ischemic colitis is primarily caused by arterial obstruction secondary to arteriosclerosis, thrombosis or embolism in the left-sided colon (1). Mesenteric venous abnormalities may also result in ischemic colitis, and lead to progressive fibrosis, calcification and obstruction of the colonic and mesenteric veins (1). Subsequently, the affected colon wall becomes thickened, which interferes with motility and may lead to subserosal calcifications and luminal stenosis (1). Ischemic colitis infrequently affects the right colon and the superior mesenteric vein (SMV).

Phlebosclerotic colitis (PC), also known as mesenteric phlebosclerosis, is a unique form of ischemic colitis with <80 reported cases in the literature (2,3). Calcifications along the wall of colon are the only unique radiographic feature, and delayed diagnosis may lead to intestinal gangrene (2,3). The present report describes a case of PC in a 56-year-old male.

## Case report

A 56-year-old male presented with a one-day history of diffuse abdominal pain, and an absence of stool passage and occasional nausea and vomiting for one week. The patient’s medical history was significant for hypertension, which had been controlled with medication for two years. In addition, the patient had used a Chinese herbal syrup and alcohol for the relief of intermittent abdominal pain for >30 years. A similar episode of diffuse abdominal pain three years previously had resulted in the patient consulting a doctor; the patient was discharged following imaging studies and conservative treatment. The study was approved by the Ethics Committee of the Institutional Review Board of Shin Kong Wu Ho-Su Memorial Hospital (Taipei, Taiwan). Written informed consent was obtained from the patient.

**Figure 1:**
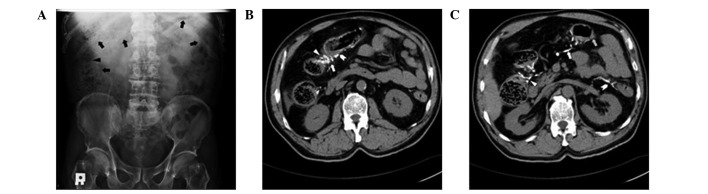
(A) Abdominal radiograph revealed fecal material in the right colon (arrowhead) with thread-like radiopaque densities representing vascular calcifications over the ascending and transverse colon (arrows). (B) CT revealed numerous serpentine calcifications (arrow) and stenosis (arrowhead) of the hepatic flexure of the colon with wall thickening (arrow tail). (C) CT also showed serpentine calcifications of the superior mesenteric vein and its tributaries of the ascending and transverse colon (arrowhead and arrow). Marked calcification of the inferior mesenteric vein and its tributaries of the descending colon was noted (arrow tail). In addition, wall thickening of the ascending, transverse and proximal descending colon, associated with poor contrast enhancement, was observed (arrow with oblique lines). CT, computed tomography.

**Figure 2:**
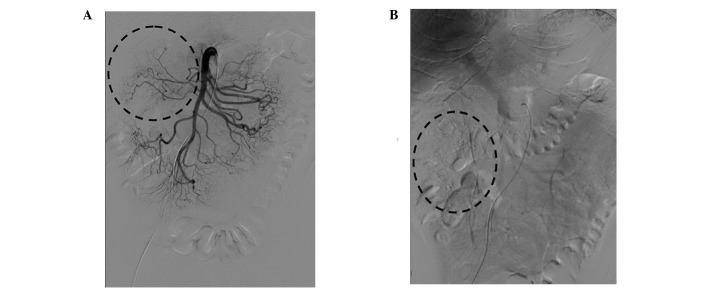
SMA angiography. (A) Arterial phase showed a patent SMA with smooth contour and normal flow; however, there was torturous narrowing and a decreased number of tributaries over the ascending and transverse colon (dashed circle). (B) Venous phase revealed markedly decreased flow in the mesenteric vein and its tributaries over the ascending and transverse colon (dashed circle). SMA, superior mesenteric artery.

Physical examination revealed stable vital signs and rebound tenderness over the right upper abdomen. Laboratory data, including biochemistry, electrolytes and a complete blood count, were all within normal limits, except for mild leukocytosis [white blood cells (WBCs), 12,600/μl; segmented neutrophils, 70.1%]. An abdominal radiograph revealed thread-like radiopaque densities in the right upper quadrant (Fig. 1A). Computed tomography (CT) revealed numerous serpentine calcifications alongside the colonic veins, which extended from the terminal ileum to the proximal descending colon, ascites, and wall thickening and luminal stenosis over the hepatic flexure (Fig. 1B and C). Compared with the previous imaging studies, the extent of the calcifications had become more severe. Angiography showed a patent superior mesenteric artery trunk; however, there was decreased arterial perfusion and absent venous return at the tributary of the ascending colon and hepatic flexure (Fig. 2).

The patient was admitted and reported increased abdominal pain and tenderness later that day, and progressive leukocytosis was observed (WBCs, 18,800/μl; segmented neutrophils, 88.0%). The patient subsequently underwent emergent surgical intervention with suspected PC and impending ischemia or gangrene of the colon.

**Figure 3:**
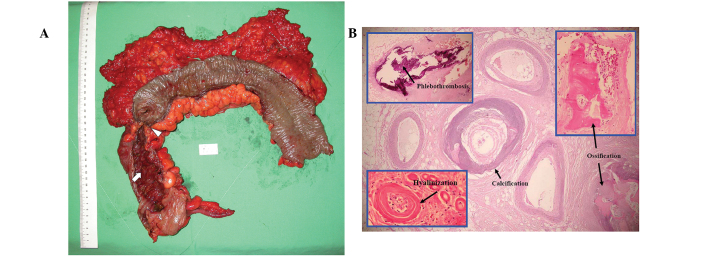
(A) The resected colon specimen was observed to have a dark purple-colored mucosal surface (arrow) with hemorrhagic spots and marked mural thickening of the colon wall, particularly the right colon. In addition, there was a segmental stenosis over the hepatic flexure (arrowhead). (B) Hematoxylin and eosin staining revealed hyalinization, calcification, ossification and thrombosis of the mesenteric veins.

Intraoperatively, ischemic and gangrenous changes involving almost the entire colon, as well as turbid ascites, were observed, and a subtotal colectomy was performed. Pathological examination revealed necrotic changes, erosion and ulceration of the colonic mucosa, in addition to hemorrhagic and edematous submucosa with inflammatory cell infiltration in the colon wall. No obvious perforation was apparent. There were numerous sclerotic veins or venules with hyalinization, calcification and areas of ossification in the intramural and extramural aspect of the colon, and a focal area of near-total occlusion of the venous lumen with ossification and thrombosis (Fig. 3).

The patient recovered well, and was discharged two weeks later. He returned to work, and has had no recurrent symptoms during the subsequent three-year follow-up.

## Discussion

The signs and symptoms of PC are nonspecific and may include abdominal pain, ileus, diarrhea and bloody stools (2). Features of PC may include mesenteric venous fibrosis, hyalinization, sclerosis and/or calcifications, ulceration of the colonic mucosa, wall thickening and luminal stricture (4,5). PC occurs primarily in individuals of Asian descent. A summary of the patient and disease characteristics is shown in Table I.

The condition is mostly neglected during the early stage of the disease, and the characteristic radiographic feature is multiple fine tortuous thread-like or serpentine mesenteric venous calcifications involving the marginal veins, vena recti and intramural tributaries (3,6). The calcifications are primarily located perpendicular to the long axis of the colon (3,6), and may extend to the vicinity of the SMV trunk (2). CT may reveal edematous wall mural thickening and luminal stricture of the colon, and angiography may show decreased venous phase perfusion. PC chiefly involves the right hemicolon, and in certain cases the lesions gradually progress in a caudal direction (3,6). In the patient described in the present report, the SMV distribution of the ascending and proximal transverse colon was affected in 2006, and three years later the lesions had extended to the proximal descending colon, suggesting a slow disease progression.

The pathogenesis of PC has not been fully elucidated (2,5). There have been seven cases of PC associated with the prolonged use of herbal medications (7,8). In addition, alcohol and chemicals, such as aristolochic acid or rolipram, have been associated with blood vessel injuries that lead to ischemic changes (7,8). The history of the patient in the present case was significant for the use of a herbal syrup for >30 years. Of note, there are numerous herbal-infused seasonings and microorganisms used in the fermenting process of foods commonly consumed in Asian countries.

**Figure 4:**
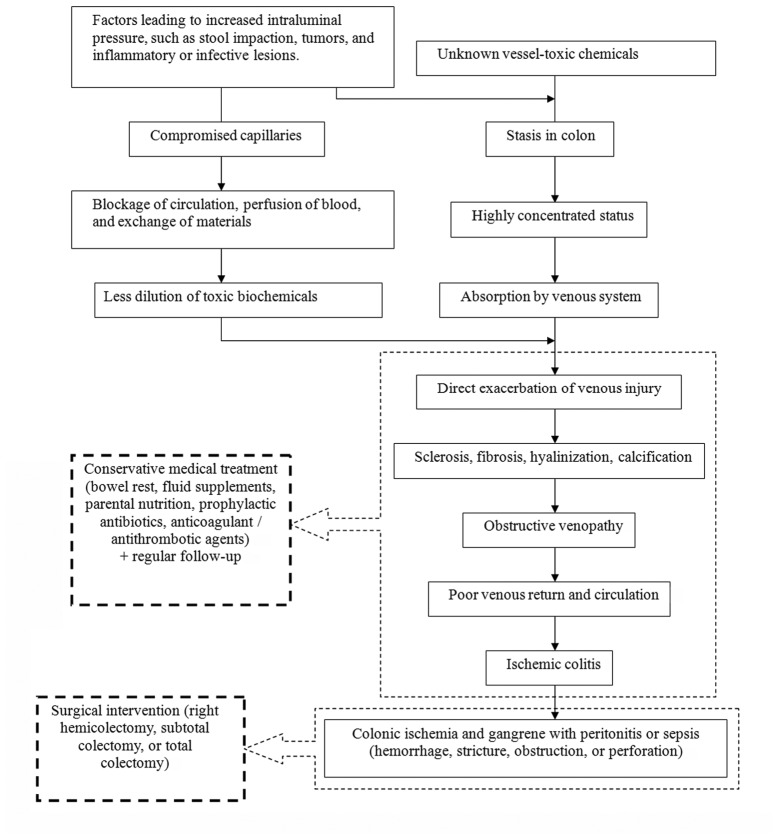
Possible algorithm of the pathogenesis and management of phlebosclerotic colitis.

It is unclear why PC primarily affects the right-sided colon; however, it has been suggested that certain toxic biochemical agents or water-soluble irritants absorbed from the ascending colon cause chronic venous damage (7,9). These substances may remain static longer in the right-sided colon, and often migrate cephalad (4). Increased intraluminal pressure in the right-sided colon may be another possible factor. Genetic susceptibility to PC has also been proposed, since almost all reported cases of PC are in individuals of Asian ancestry (4). Based on our review of the literature, an algorithm of the possible pathogenic mechanism is shown in Fig. 4.

The management of PC should be based on the severity of the disease. In addition, the extent and duration of blood supply deprivation, as well as the degree of the intestinal injury, should be considered when selecting between surgical and conservative treatment. Previously, the majority of patients underwent surgical intervention; however, at present, conservative management with close follow-up is preferred if there are no signs of bowel compromise (10). Hemicolectomy or subtotal or total colectomy is considered curative therapy with a relatively good prognosis (10).

PC is a rare and potentially life-threatening condition that is most frequently observed in Asia, and diagnosis is made by the presence of serpentine calcifications on imaging studies. Management depends on the severity of disease, ranging from close follow-up to prompt surgical intervention.

## Figures and Tables

**Table I:** Demographics and characteristics of patients with phlebosclerotic colitis reported from 1991 to 2011.

Demographic/characteristic	Value	%
Country or area (n=69)		
Japan	52	75.4
Taiwan	12	17.4
Hong Kong	2	2.9
Korea	3	4.3
Gender (n=63)		
Male	34	54.0
Female	29	46.0
Age, years (n=63)		
Median (range)	59 (33–77)	NA
Symptoms and signs (n=60)		
Pain	45	75.0
Diarrhea	26	43.3
Ileus	17	28.3
Nausea/vomiting	17	28.3
Positive stool occult blood	12	20.0
Constipation	8	13.3
Palpable mass	3	5.0
Fever	3	5.0
Body weight loss	3	5.0
Fatigue	1	1.7
Location of lesions (n=62)^a^		
Limited to right colon (cecum and ascending colon)	4	6.5
Continuously extended to transverse colon	29	46.8
Continuously extended to left colon (descending and sigmoid colon)	27	43.5
Continuously extended to rectum	2	3.2
Management (n=64)		
Surgical intervention	33	51.6
Conservative treatment and subsequent surgery	5	7.8
Conservative treatment only	26	40.6
Procedure (n=38)^b^		
Right hemicolectomy	12	31.6
Subtotal colectomy	26	68.4

a In all cases the right colon was involved.

b In two cases a laparoscopic procedure was performed. Two patients succumbed to septic shock on postoperative day 3.

NA, not applicable.
